# Supplementary material for: Performance Characteristics of Zeno Trap Scanning DIA for Sensitive and Quantitative Proteomics at High Throughput
Source: Proteomics. 2025 Dec 26;26(1):68–81. doi: 10.1002/pmic.70093 (PMC12809002; doi:10.1002/pmic.70093)
Supplement: Supplementary file 1 — Supporting File: pmic70093‐sup‐0001‐SuppMat.pdf. [file PMIC-26--s001.pdf]

Supplementary Figures

-

Performance Characteristics of Zeno Trap Scanning DIA for Sensitive and Quantitative Proteomics at High Throughput

Ludwig R. Sinn[1], Ziyue Wang[1], Claudia P. Alvarez[2], Anjali Chelur[2], Ihor Batruch[2], Patrick Pribil[2], Daniela Ludwig[1], Stephen Tate[2✉], Jose Castro-Perez[2], Christoph B. Messner[3], Vadim Demichev[1] and Markus Ralser[1,4,✉]

[1]Department of Biochemistry, Charité – Universitätsmedizin Berlin, Berlin, Germany
[2]Sciex, Concord, Canada
[3]Precision Proteomics Center, Swiss Institute of Allergy and Asthma Research (SIAF), University of Zurich, Davos, Switzerland
[4]The Wellcome Centre for Human Genetics, Nuffield Department of Medicine, University of Oxford, United Kingdom

[✉]Correspondence to: markus.ralser@charite.de and stephen.tate@sciex.com

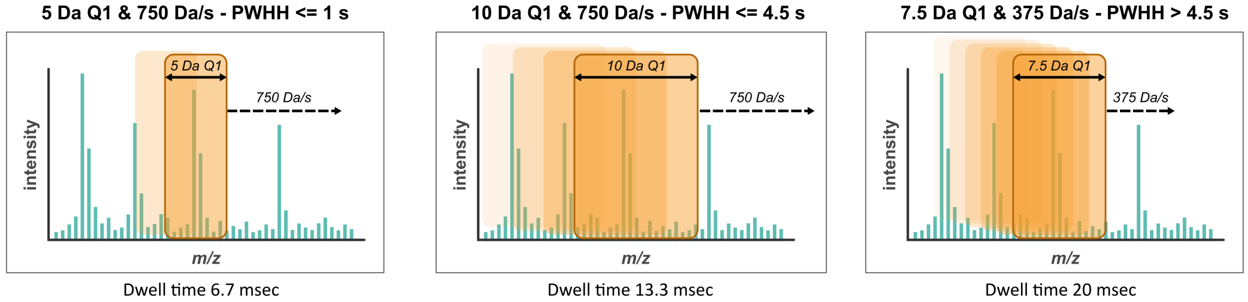

Fig. S1: Scheme on scanning DIA precursor isolation for three ZT Scan DIA methods

One can choose from either of the three ZT Scan DIA methods that differ in their Q1 isolation width (orange boxes) and Q1 scan speeds (dashed arrow) to arrive at individual performance optima depending on a peptide's chromatographic peak width at half height (PWHH). Fragment ion intensities depend on the time - the dwell time - a precursor ion is isolated across multiple subsequent sliding Q1 isolation windows (i.e., being covered by orange boxes; each box here corresponds to ~3.34 msec). Precursor ion dwell times for ZT Scan DIA methods are indicated beneath each panel.

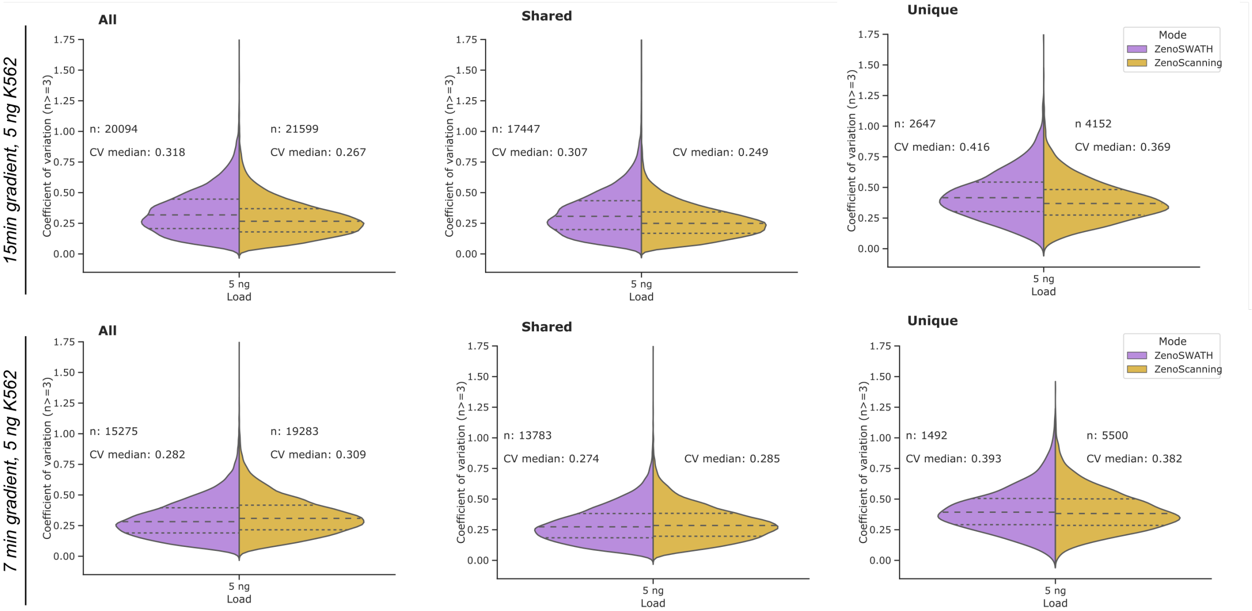

Fig. S2: Quantitative precision of precursor quantities from eleven low-load acquisitions comparing ZT Scan DIA with Zeno SWATH DIA

Each panel illustrates the distribution of coefficients of variation for quantified precursors (observed at least three times) per method, in set categories of either all precursors detected per method, being shared between methods, or being unique to each method. Numbers of observations and medians are indicated per method and across categories. The color code is indicated in the right-most panels.

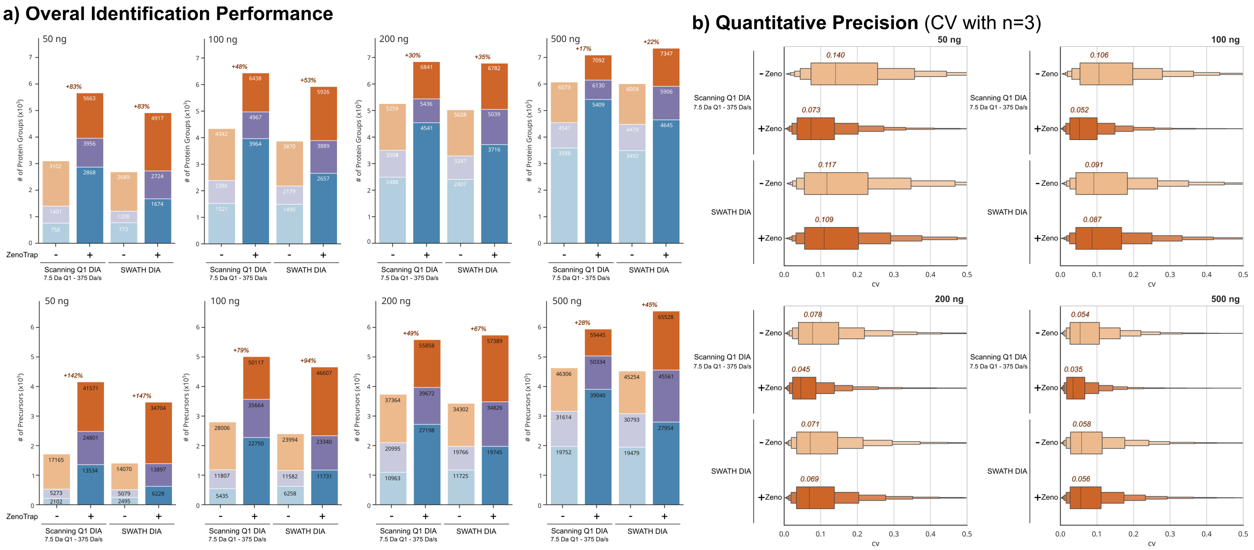

Fig. S3: Effect of Using Zeno Trap Ion Trapping for Consecutive-window and Scanning Q1 SWATH DIA

K562 human proteome digest standard was acquired using a 15-min active gradient at 50, 100, 200 and 500 ng sample load. Panels in (a) display the number of unique protein groups and precursor ions filtered to 1% FDR each, from triplicate injections and while varying the loaded sample amounts (average: orange, ≤20% CV: purple, ≤10% CV: blue; SWATH or Scanning DIA, and Zeno SWATH or ZT Scan DIA are shown with lower and higher saturation, respectively). The corresponding numbers for each category are indicated just below the top of each bar. Percent average gains from non-Zeno to Zeno trap methods are indicated on top of each DIA method pair. Note that for Scanning Q1 methods, only the 7.5 Da Q1 - 375 Da/s setup could be compared. Panels in (b) illustrate the precision of derived protein group quantities with consistent identification (n=3) shown as distributions of coefficients of variation (CV) across all sample loads (indicated in the top right of each subpanel) as Boxenplots. Medians and 50% inter-quartile ranges (IQRs) are highlighted by the central box while each quantile further outwards represents half of the remaining fraction. The medians for each method and sample load are indicated above each IQR.

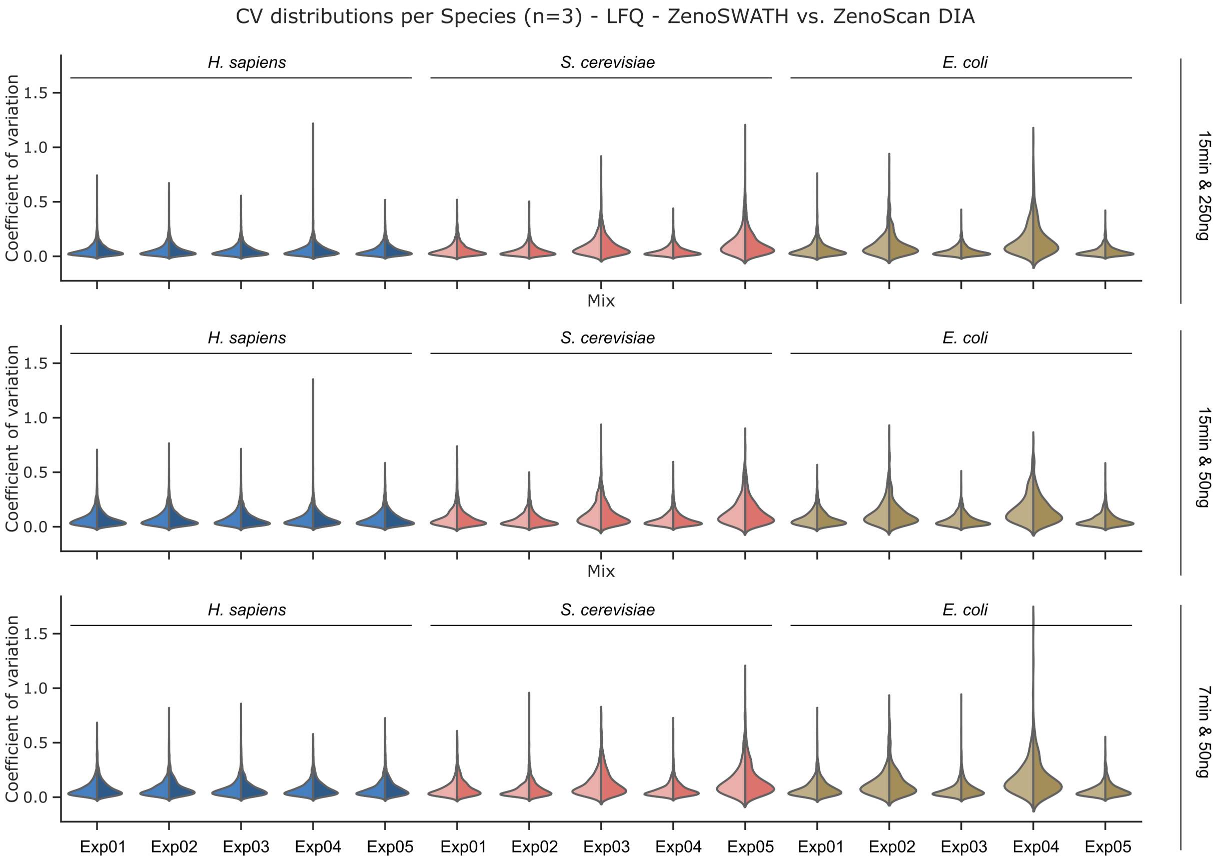

Fig. S4: Quantitative precision for precursor identifications in LFQ bench experiment comparing ZT Scan DIA with Zeno SWATH DIA

Violin plots illustrate the distribution of coefficients of variation for precursors observed at least three times, for each experimental mix (refer to Fig. 4a for exact ratios), and per species (human: blue, yeast: red, bacteria: brown). As indicated on the right side, each panel row represents an active gradient (15 or 7 min) and the total amount of loaded sample per LC-MS acquisition (250 or 50 ng).

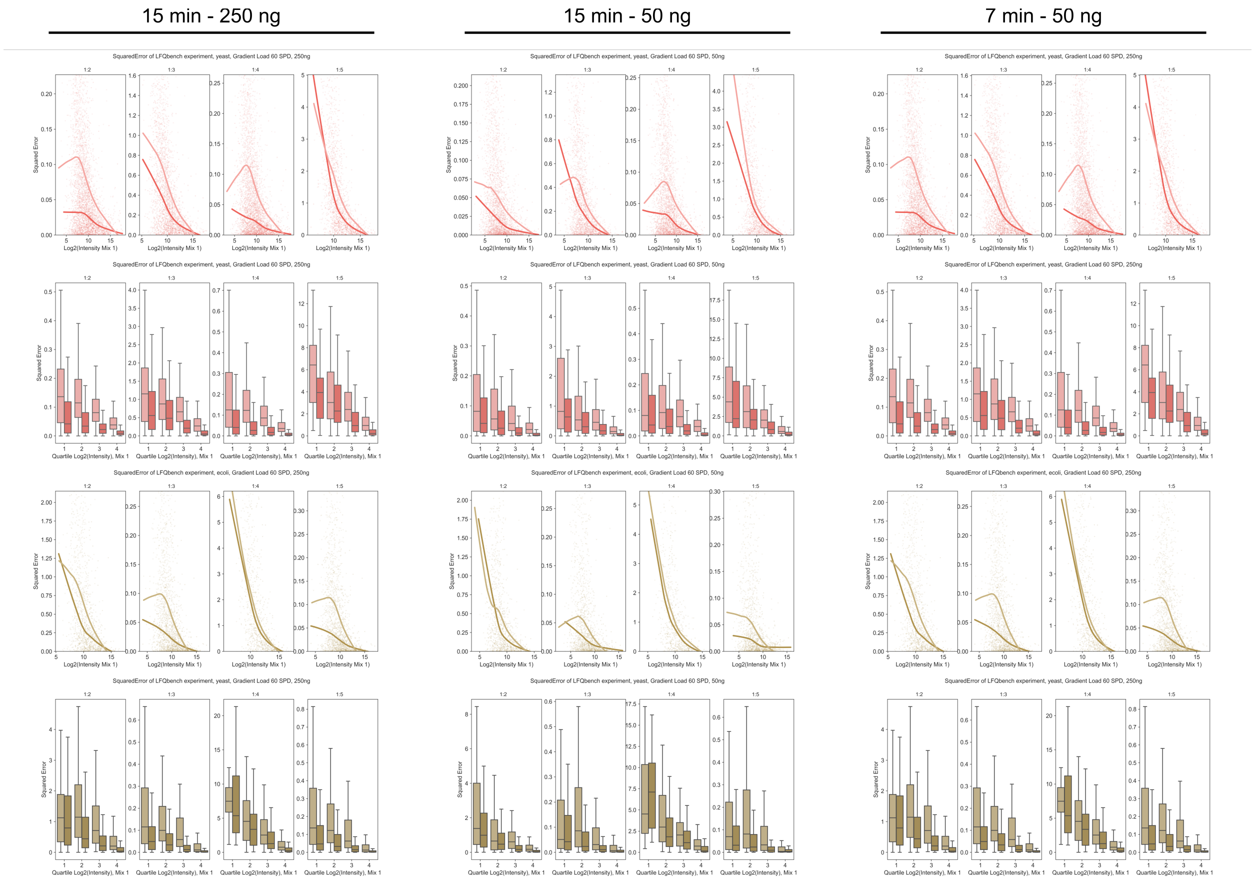

Fig. S5: Quantitative accuracy for protein group identifications in LFQ-bench experiment comparing ZT Scan DIA with Zeno SWATH DIA

The quantitative accuracy was assessed based on the difference (as squared error) of a measured quantity to its expected quantity (given the defined mixing ratios). Each column illustrates the experimental condition applied with regard to active chromatographic gradient and total sample load. In each column, each row consists of four panels that indicate the quantitative accuracy for each of the four experimental mixes compared to the standard mix (i.e., to experimental mix 1, with equal relative ratios of all tested proteomes). Datapoints in scatterplots were fitted using LOESS regression to highlight the trends. Boxplots show the same trends across log2 intensity quartiles (boxes cover 50% of the data while the whiskers span 1.5x the IQR; outliers are not shown). Darker colouring highlights the performance of ZT Scan DIA while the lighter hue stands for the corresponding one for Zeno SWATH DIA.

Red colouring indicates errors of yeast protein groups while brown colouring indicates those of *E. coli*.

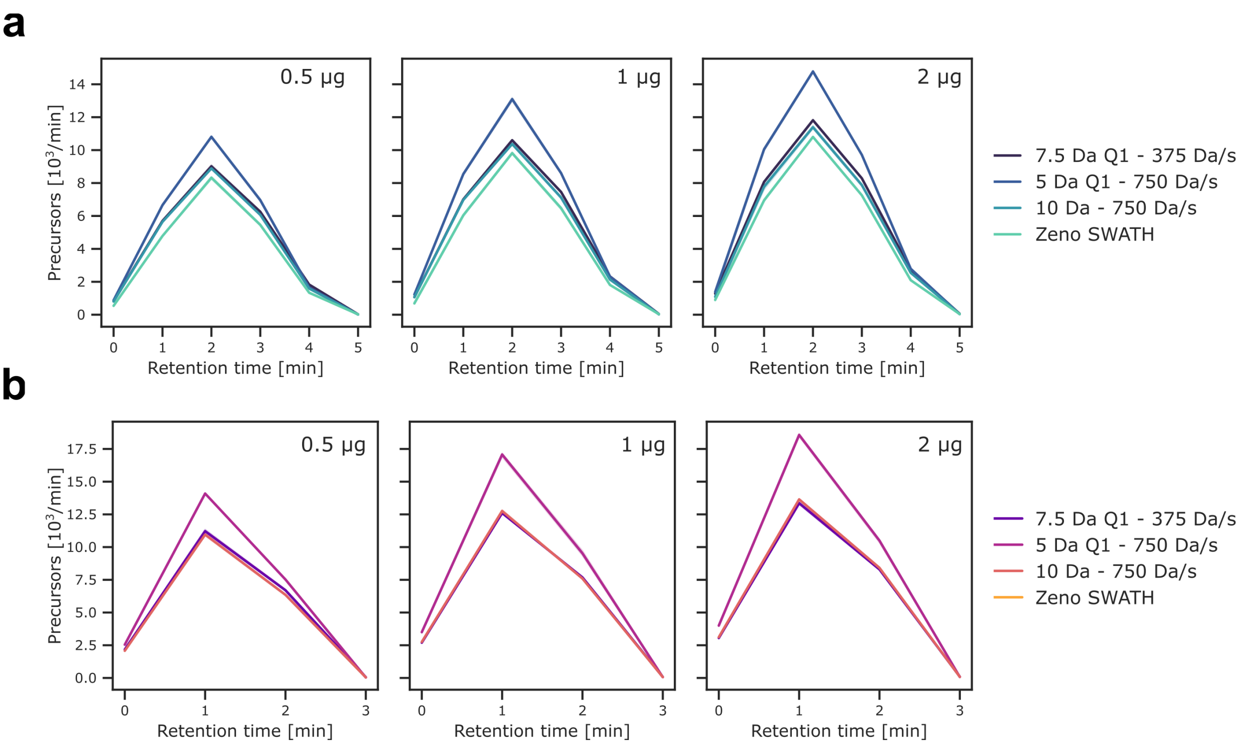

Fig. S6: Precursors per minute in analytical flow rate chromatography experiment
Precursors per minute are plotted for each sample load of HEK cell proteome digest, pooled for triplicate injections, for the 5.25-min active gradient (a) and the 3.1-min active gradient (b).

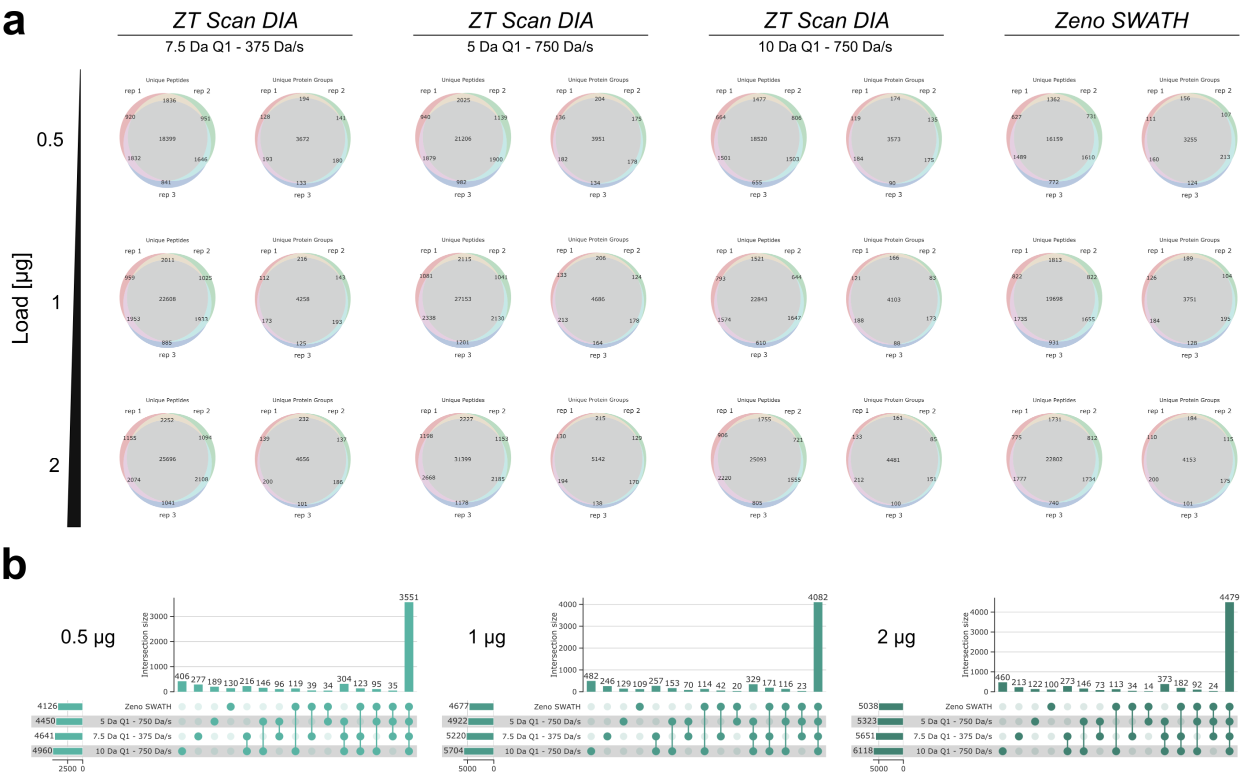

Fig. S7: Set overlaps between replicates and both MS methods for 5.2 min active analytical gradient

a) Venn diagrams showcasing overlaps of Peptides (left) or Protein groups (right) within replicates of a loading amount and LC-MS method combination (indicated above). b) Upset Plot illustrating the overlaps of peptide identifications between different LC-MS methods (3x ZT Scan DIA and Zeno SWATH) and sample loading amounts (per panel), pooled for replicates.

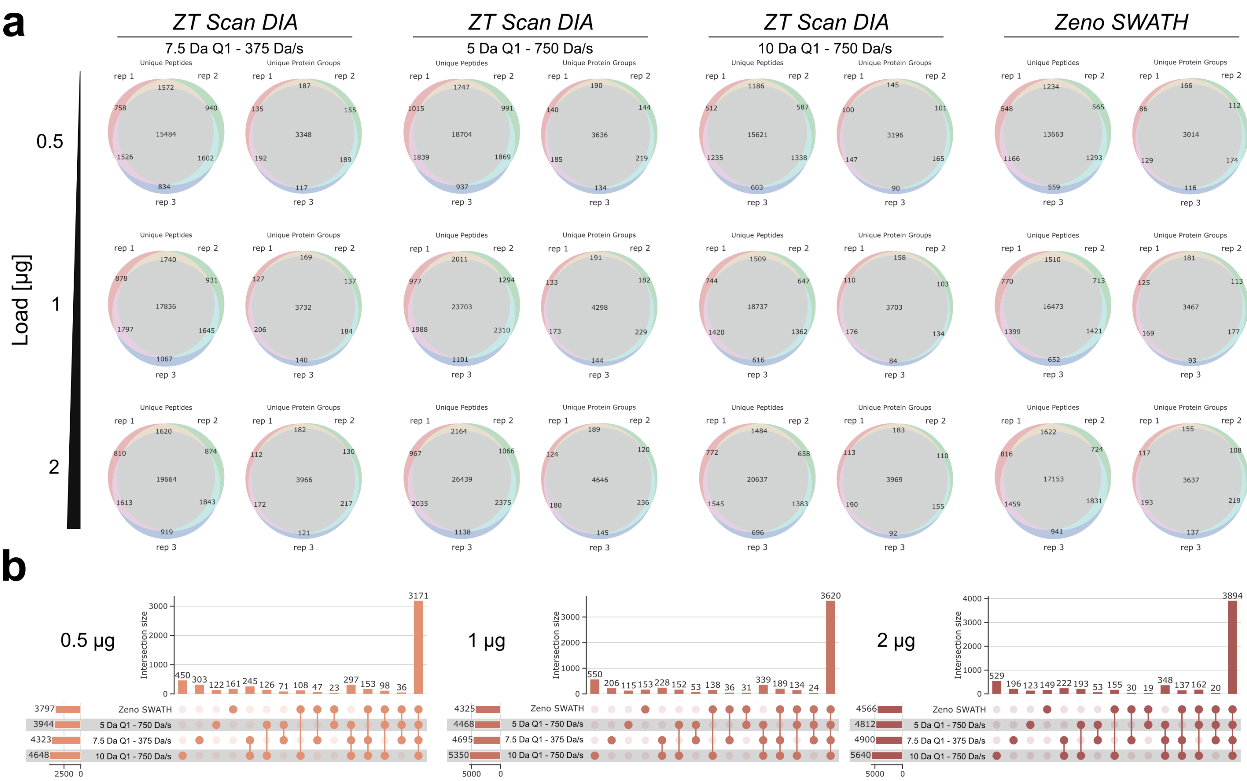

Fig. S8: Set overlaps between replicates and both MS methods for 3.1 min active analytical gradient

a) Venn diagrams showcasing overlaps of Peptides (left) or Protein groups (right) within replicates of a loading amount and LC-MS method combination (indicated above). b) Upset Plot illustrating the overlaps of peptide identifications between different LC-MS methods (3x ZT Scan DIA and Zeno SWATH) and sample loading amounts (per panel), pooled for replicates.

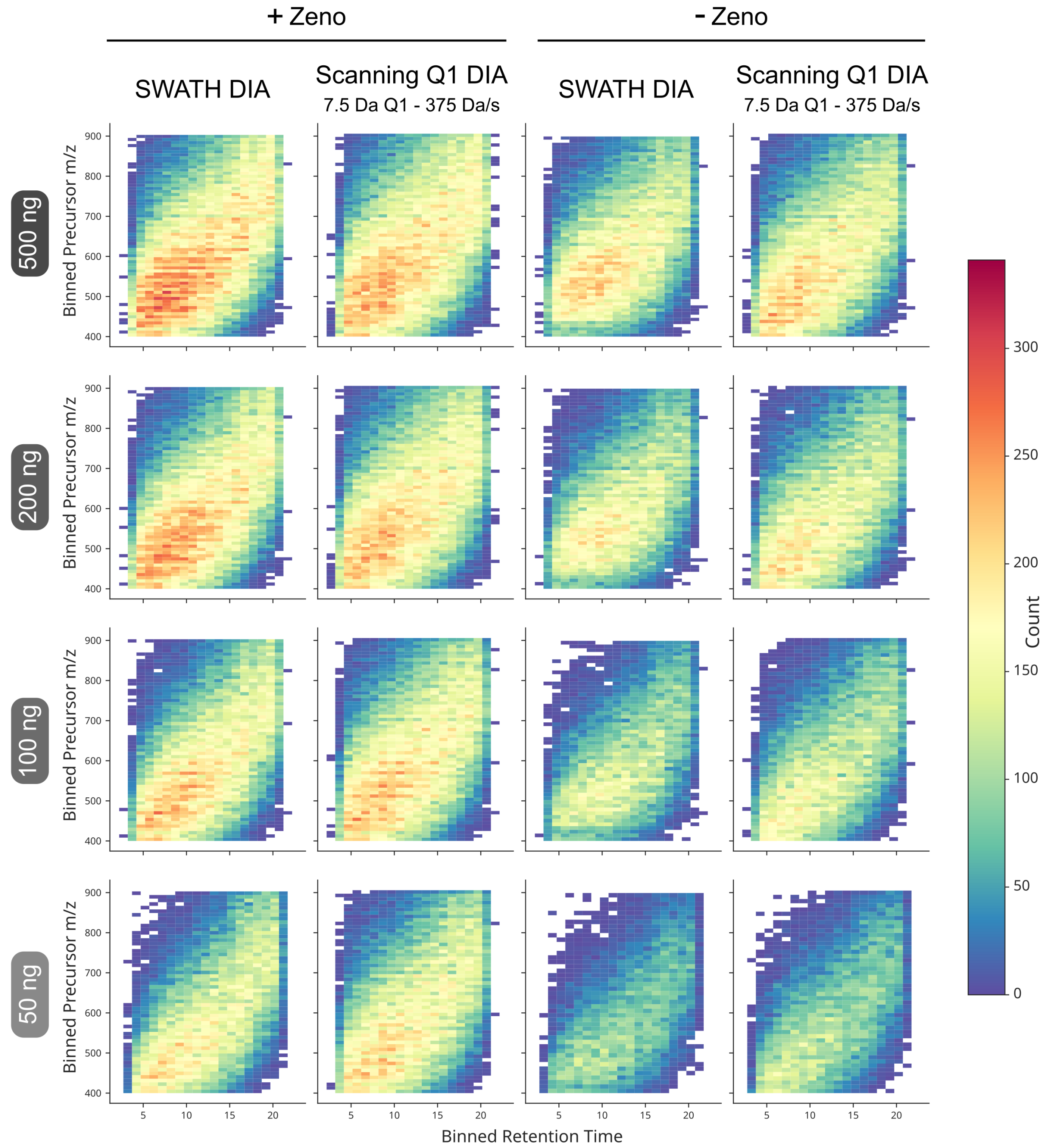

Fig.S9: Precursor Identification Performance Across the m/z and Time Domain in SWATH and Scanning Q1 DIA +/- Zeno Trap

Proteomic data for a human K562 cell line proteome tryptic digest standard was acquired using a 15-min active gradient at 50, 100, 200 and 500 ng sample load. Identified precursor ions filtered to 1% FDR each, were counted over m/z and retention time bins (500 x 499) while summing over triplicate injections for Zeno SWATH, ZT Scan DIA, SWATH, and Scanning DIA methods. Precursor counts per bin are highlighted by color as depicted on the right.

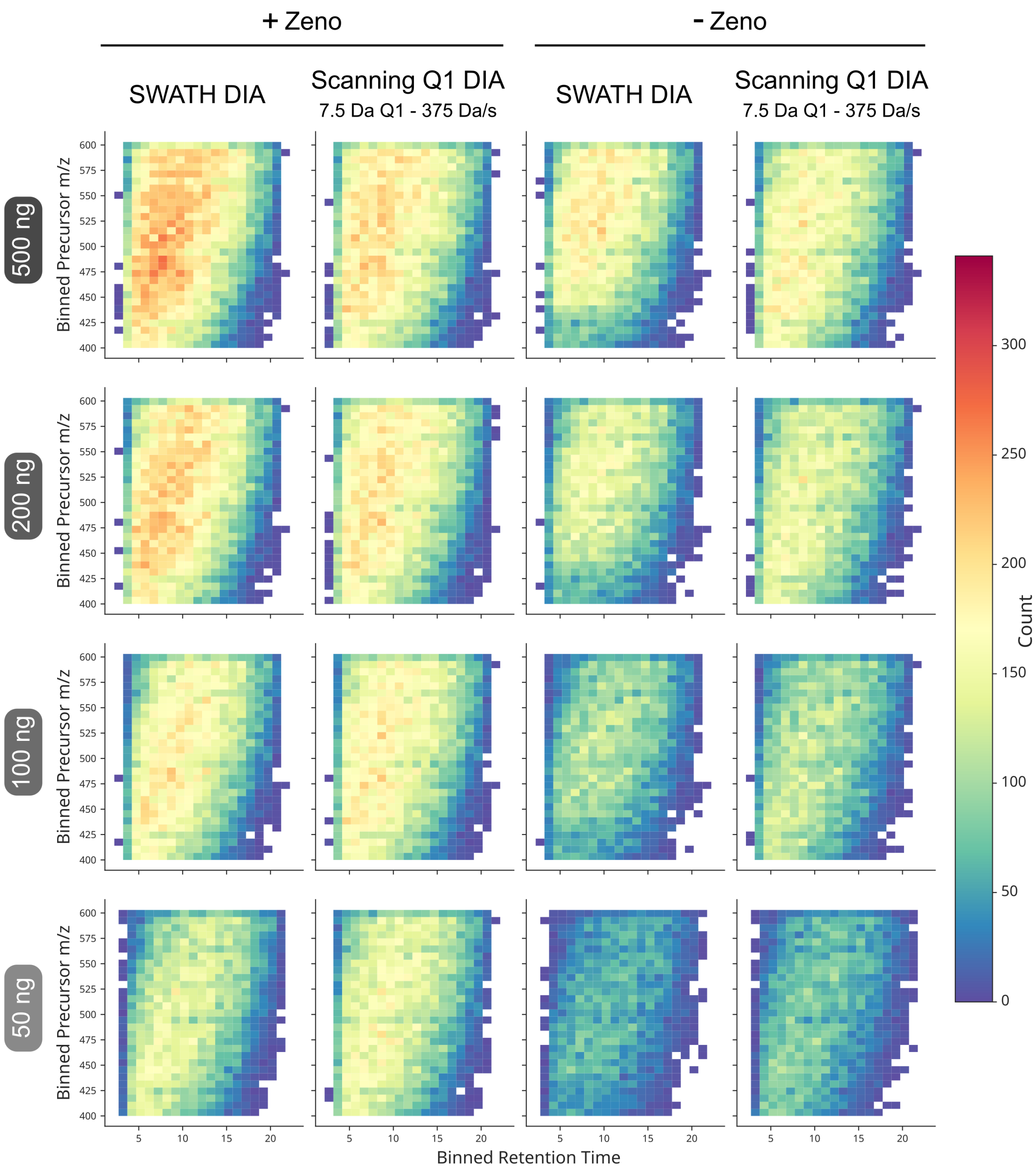

Fig.S10: Precursor Identification Performance Across the m/z and Time Domain of Highest Density in SWATH and Scanning Q1 DIA +/- Zeno Trap

Proteomic data for a human K562 cell line proteome tryptic digest standard was acquired using a 15-min active gradient at 50, 100, 200 and 500 ng sample load on the ZenoTOF7600+ instrument. Identified precursor ions filtered to 1% FDR each, were counted over m/z (400 - 600) and retention time bins (500 x 499) while summing over triplicate injections for Zeno SWATH, ZT Scan DIA, SWATH, and Scanning DIA methods. Precursor counts per bin are highlighted by color as depicted on the right.

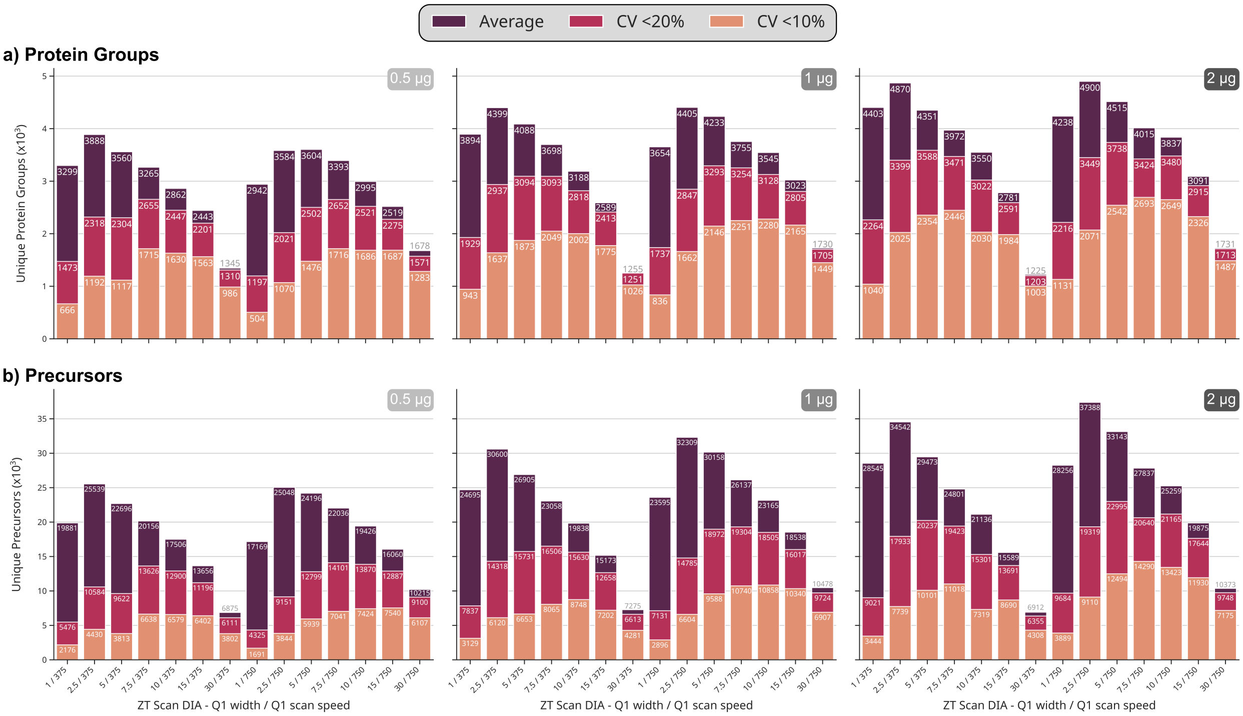

Fig. S11: Dependencies of Q1 Isolation Width and Scan Speed for ZT Scan DIA Suggests Direction for Future Developments

Assessment of ZT Scan DIA's performance in dependence of set Q1 isolation window width (1 / 2.5 / 5 / 7.5 / 10 / 15 / 30 Da) and Q1 scan speed (375 / 750 Da/s) on an analytical flow rate HPLC setup. Panel (a) shows identifications of protein groups while panel (b) displays precursors at increasing sample loads of 0.5 / 1 / 2 µg human proteome digest (from left to right) using a 3.1-minute active gradient. Three categories are shown in colour: average identifications from triplicates, identifications below or equal to a CV of 0.2, and of 0.1 (from top to bottom, n=3), respectively. Numbers for each category are indicated below a bar's top when possible. The data was filtered to 1% FDR on the respective level.
